# Supplementary material for: End Sequence Analysis Toolkit (ESAT) expands the extractable information from single-cell RNA-seq data
Source: Genome Res. 2016 Oct;26(10):1397–410. doi: 10.1101/gr.207902.116 (PMC5052061; doi:10.1101/gr.207902.116)
Supplement: Supplemental Material [file supp_gr.207902.116_Supp_Materials.tar.gz › ESAT-esat_v0.1/doc/html/classjaligner_1_1_smith_waterman_gotoh.html]

ESAT: jaligner.SmithWatermanGotoh Class Reference


ESAT


- Main Page
- Related Pages
- Packages
- Classes
- Files

- Class List
- Class Index
- Class Hierarchy
- Class Members

All Classes Namespaces Files Functions Variables Enumerator Pages

- jaligner
- SmithWatermanGotoh

Static Public Member Functions |
List of all members

jaligner.SmithWatermanGotoh Class Reference

|  |  |
| --- | --- |
| Static Public Member Functions | |
| static Alignment | align (Sequence s1, Sequence s2, Matrix matrix, float o, float e) |
|  | |

## Detailed Description

An implementation of the Smith-Waterman algorithm with Gotoh's improvement for biological local pairwise sequence alignment.

**Recursive definition:**

- **Base conditions:**
  - `V(0, 0) = 0`
  - `V(i, 0) = E(i, 0) = Wg + iWs`
  - `V(0, j) = F(0, j) = Wg + jWs`
- **Recurrence relation:**
  - `V(i, j) = max{E(i, j), F(i, j), G(i, j)}`, where:
  - `G(i, j) = V(i - 1, j - 1) + similarity(Si, Tj)`
  - `E(i, j) = max{E(i, j - 1) + Ws, V(i, j - 1) + Wg + Ws}`
  - `F(i, j) = max{F(i - 1, j) + Ws, V(i - 1, j) + Wg + Ws}`

Author
:   Ahmed Moustafa (ahmed.nosp@m.@use.nosp@m.rs.sf.nosp@m..net)

## Member Function Documentation

|  |  |  |  |
| --- | --- | --- | --- |
| static Alignment jaligner.SmithWatermanGotoh.align | ( | Sequence | *s1*, |
|  |  | Sequence | *s2*, |
|  |  | Matrix | *matrix*, |
|  |  | float | *o*, |
|  |  | float | *e* |
|  | ) |  |  |

static

Logger Aligns two sequences by Smith-Waterman (local)

Parameters
:   |  |  |
    | --- | --- |
    | s1 | sequene #1 (Sequence) |
    | s2 | sequene #2 (Sequence) |
    | matrix | scoring matrix (Matrix) |
    | o | open gap penalty |
    | e | extend gap penalty |

Returns
:   alignment object contains the two aligned sequences, the alignment score and alignment statistics

See also
:   Sequence
:   Matrix

---

The documentation for this class was generated from the following file:

- java/jaligner/SmithWatermanGotoh.java


---

Generated on Fri Aug 15 2014 15:11:05 for ESAT by  

 1.8.7
